# Adaptive optimization by 6 DOF robotic couch in prostate volumetric IMRT treatment: rototranslational shift and dosimetric consequences

**DOI:** 10.1120/jacmp.v16i5.5525

**Published:** 2015-09-08

**Authors:** Silvia Chiesa, Lorenzo Placidi, Luigi Azario, Gian Carlo Mattiucci, Francesca Greco, Andrea Damiani, Giovanna Mantini, Vincenzo Frascino, Angelo Piermattei, Vincenzo Valentini, Mario Balducci

**Affiliations:** ^1^ Department of Radiation Oncology Università Cattolica del Sacro Cuore Rome Italy; ^2^ Department of Medical Physics Università Cattolica del Sacro Cuore Rome Italy

**Keywords:** IMRT, prostate cancer, robotic couch, setup correction, dosimetric impact of 6 DOF patients' position correction

## Abstract

The purpose of this study was to investigate the magnitude and dosimetric relevance of translational and rotational shifts on IGRT prostate volumetric‐modulated arc therapy (VMAT) using Protura six degrees of freedom (DOF) Robotic Patient Positioning System. Patients with cT3aN0M0 prostate cancer, treated with VMAT simultaneous integrated boost (VMAT‐SIB), were enrolled. PTV2 was obtained adding 0.7 cm margin to seminal vesicles base (CTV2), while PTV1 adding to prostate (CTV1) 0.7 cm margin in all directions, except 1.2 cm, as caudal margin. A daily CBCT was acquired before dose delivery. The translational and rotational displacements were corrected through Protura Robotic Couch, collected and applied to the simulation CT to obtain a translated CT (tCT) and a rototranslated CT (rtCT) on which we recalculated the initial treatment plan (TP). We analyzed the correlation between dosimetric coverage, organs at risk (OAR) sparing, and translational or rotational displacements. The dosimetric impact of a rototranslational correction was calculated. From October 2012 to September 2013, a total of 263 CBCT scans from 12 patients were collected. Translational shifts were <5mm in 81% of patients and the rotational shifts were $<2^{\circ }$ in 93% of patient scans. The dosimetric analysis was performed on 172 CBCT scans and calculating 344 VMAT‐TP. Two significant linear correlations were observed between yaw and the V20 femoral heads and between pitch rotation and V50 rectum (p<0.001); rototranslational correction seems to impact more on PTV2 than on PTV1, especially when margins are reduced. Rotational errors are of dosimetric significance in sparing OAR and in target coverage. This is relevant for femoral heads and rectum because of major distance from isocenter, and for seminal vesicles because of irregular shape. No correlation was observed between translational and rotational errors. A study considering the intrafractional error and the deformable registration is ongoing.

PACS number: 87.55.de

## I. INTRODUCTION

Technical advances in radiotherapy have become prevalent over the last years. Intensity‐modulated radiotherapy (IMRT) and volumetric‐modulated arc therapy (VMAT) allow us to deliver steep dose gradients at the PTV, reducing exposure to adjacent normal tissues.

Temporal changes in the patient's anatomy, inter/intrafractional organ motion or setup errors can determine suboptimal dose's distribution on the tumor or increase dose to the critical structures; therefore, additional margins are added to the clinical target volume (CTV).^(1)^

More recently, the advent of image‐guided radiation therapy (IGRT) has enabled us to achieve sophisticated three‐dimensional volume and soft‐tissue contrast information.^2^, ^3^ They include mobile in‐room computed tomography (CT) scanners,^(4)^ CT on rails,^5^, ^6^ B‐mode ultrasound systems,^(6)^ tomotherapy,^(6)^ and kilovoltage^7^, ^8^ and megavoltage^2^, ^9^ cone‐beam CT scanners (CBCT), widely in use currently.

In the past, geometric corrections were often limited to linear identified shifts, as well as sometimes table rotation (yaw), while the other two angular corrections (pitch and roll) were frequently ignored. A wide range of online position correction dosimetric effects have been observed and it is increasingly becoming a field of interest.^10^, ^11^, ^12^, ^13^, ^14^ Several authors studied the target coverage of the prostate after applying translational position correction. They concluded that, on average, the target dose coverage after position correction is higher than without correction, but lower than in the treatment plan, and explained the remaining underdosage by rotations, not included in the correction, and by deformations. Therefore, other investigators simulated the relevance of rotation and its impact on dosimetric coverage, but the relationship among prostate rotation, dosimetric coverage, and rotation management have yet to be defined.^14^, ^15^

Similarly to the majority of the studies that have dealt with setup errors they reported only about translational errors. Limited data exist regarding the magnitude and relevance of rotational errors.^16^, ^17^, ^18^ Not all standard treatment tables allow rotational corrections; therefore, strategies for six‐degrees‐of‐freedom (DOF) corrections have been discussed and included the use of a combination of collimator, gantry, and/or the implementation of a robotic couch.^19^, ^20^, ^21^, ^22^, ^23^ Soete et al.^(21)^ have proven that this 6 DOF patient setup correction improves the patient setup accuracy, and other authors reported experiences about the relevance of rotational shift when patients received high precision radiotherapy.^14^, ^15^, ^16^, ^17^, ^18^, ^19^, ^20^, ^21^, ^22^

In August 2012, Protura Robotic Patient Positioning System (CIVCO Medical Solutions, Coralville, IA) was installed on Varian Linac 2100 with on‐board cone‐beam CT system. Several studies were designed to evaluate the robotic couch practice in different tumor sites or radiation techniques. In this article, we analyzed the role of 6 DOF correction on geometric and dosimetric variability in prostate VMAT treatment.

## II. MATERIALS AND METHODS

Patients affected by prostate cancer (cT3aNoMo), having undergone VMAT‐SIB, were enrolled in this study. For low nodal involvement risk, CTVs included only prostate and seminal vesicles base.

### A. Treatment plan

Patients underwent a non‐contrast CT simulation scan with 1.25 mm slice thickness in supine position with a conventional head support; arms were positioned on the thorax and the knees and ankles were supported with the dual leg (CIVCO Medical Solutions) to ensure reproducible position. The clinical target volumes (CTV) were contoured by a radiation oncologist on each slice of CT images. The PTV2 was obtained adding 0.7 cm margin to seminal vesicles base (CTV2), while PTV1 adding 0.7 cm margin in all directions, except 1.2 cm as caudal margin, to prostate (CTV1). The bladder, the rectum, the bowel, and the femoral heads were also contoured. The prescription doses were 80 Gy (2 Gy/day) to PTV1 and 72 Gy to PTV2 (1.8 Gy/day). The VMAT‐SIB plans consisted of two arcs, and dose optimizations were performed using Varian Eclipse external beam planning system, version 8.9 (Varian Medical Systems, Palo Alto, CA) with the AAA algorithm for dose calculation and PRO2 optimization system (referent treatment plan=refTP).

According to the our standard clinical procedure and protocol,^24^, ^25^ the first CTVs delineation was adapted to organ motion considering the position of prostate and seminal vesicle base through CBCT scans acquired during the first five days (new CTV1 and new CTV2). We performed a second treatment plan (replanning treatment plan=refTP) on new PTVs defined as new CTV1 and new CTV2+0.3 cm of margins.

### B. Treatment delivery

After patients positioning, a CBCT study was performed every second day, excepting a daily scan during the first and the last treatment week, since major displacements are expected due to patients compliance (first week) or to acute toxicity (last week). CBCT images, by manual or automatic 3D match, were digitally overlaid with the reference CT images and patient position was corrected. Translational and rotational values relative to the applied correction were collected.

The Protura robotic couch allowed a 6 DOF (X,Y,Z, pitch, roll and yaw) patient positioning correction. The couch top is mounted on Varian Clinac pedestal and the pedestal movement is independent from the upper Protura. In this couch top, replacement is based on parallel cinematics system, where all the actuators move simultaneously. Position verification is performed without external optical system. It is essential, each time, to select the center of rotation, the pivot point, always coinciding with the patient's isocenter. The table positioning is controlled by a remote computer outside the treatment room and an internal touch screen monitor. The integration with Varian's Motion Management Interface enables us to automatically insert the shift identified.

### C. Adaptation of treatment plans to geometrical displacements

MIM Maestro 5.5.2 software (MIM Software Inc., Cleveland, OH, USA) was used to consider, simultaneously, rotations and translation on all axes.

To evaluate the dosimetric differences between the 6‐ or 3‐DOF corrections, every CBCT of each fraction were rigidly registered to the planning CT scan, creating a new CT on MIM software. According to a validated coordinate conversion system (between CT simulation, robotic couch and MIM software), the first CT shift was obtained applying only translational values (translated $\text{CT}=t_{n}\text{CT}$, where n=number of fractions), and the second one applying also the rotational values to obtain a rototranslated CT (${\text{rt}}_{n}\text{CT}$) which represented the patients' position after 6DOF correction. Then the refTP and replTP plans were copied and calculated on $t_{n}\text{CT}$ and ${\text{rt}}_{n}\text{CT}$, producing translated plans ($t_{n}\text{TP}$) and rototranslated plans (${\text{rt}}_{n}\text{TP}$), respectively. The daily doses delivered were calculated.

### D. Geometrical and dosimetric analysis

Setup and organ motion displacements were defined as deviation between expected simulation CT and the actual position acquired mean of the CBCT before treatment. Errors were calculated separately for all three axes: x‐, y‐, and z‐ axis, both translational and rotational. The mean and standard deviation (SD) were computed for each kind of translational and rotational shift for all the patients. For all the 6 DOF, average Σ and standard deviation (SD) σ were computed for each patient and for all the population. The correlation factor R, which indicates how well data points fit a statistical model (in that case, linear), was calculated to investigate the relation between the magnitude of translational and rotational shift. Using the Van Herk formula and margins criteria determination,^(26)^ the Σ and σ values previously calculated were used to obtain the population PTV margins.

**Figure 1:**
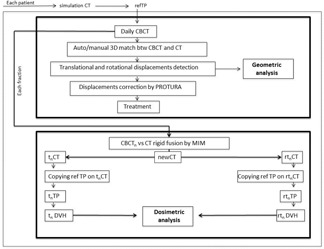
Workflow of the geometric and dosimetric analysis.

Regarding dosimetric analysis, patients with a CBCT acquisition according to our schedule were considered. The dosimetric impact of a 6D patient positioning correction was evaluated comparing refTP or replTP and $t_{n}\text{TP}/{\text{rt}}_{n}\text{TP}$. This workflow is represented in Fig. 1.

Through a homemade program, PTV1 and PTV2 coverage (volume receiving 95% or 105% of the dose [V95%‐V105%]), V50 Gy rectum (volume receiving 50 Gy), V20Gy of femoral heads (volume receiving 20 Gy), V45 bowel (volume receiving 45 Gy), and the maximum bladder dose were calculated. The dosimetric coverage and sparing parameters in translational or rotational shift (correlation factor R and R^2^ and *p* level) were analyzed. The dosimetric impact of a translational correction was obtained as the difference between $t_{n}\text{TP}$ and refTP(tTP−refTP=A), while rototranslational one as the difference between ${\text{rt}}_{n}\text{TP}$ and refTP(rtTP−refTP=B). Finally, the impact of a rotational correction through the dosimetric difference was calculated as C=|A−B|.

The dosimetric impact of the 6 DOF corrections was also analyzed, comparing dosimetric differences (C) between planning (Cpl) and replanning (Crepl) phase to evaluate the new PTVs dosimetric impact.

## III. RESULTS

The Protura System is user friendly, allowing for a fast learning curve and the possibility to be integrated into our daily clinical practice. IGRT workflow was not affected, both in terms of time and department human resources. From October 2012 to September 2013, 147 patients benefited from the 6D repositioning system and 90 were included in this prospective study. Twelve of them were affected by prostate cancer and treated by VMAT‐SIB. Compliance was of 92.3%; only one patient was not able to maintain the position because of neuropsychological disorders. Two hundred and sixty‐three CBCTs were obtained and analyzed in terms of geometrical results, while 172 CBCTs were used to calculate 172 tPT and 172 trPT (344 total TP) in terms of dosimetric analysis.

### A. Geometric analysis

**Figure 2:**
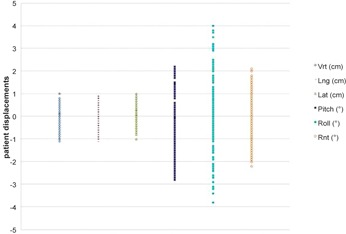
Distribution of translational and rotational patient setup errors.

On 263 3D match CT‐CBCT, the mean±SD interfraction displacement in vertical, longitudinal, and lateral directions was −1.4±4.1mm,−1.4±4.2mm, and 0.5±3.6 mm, respectively. The mean (±SD) interfraction rotations were: $\text{pitch}=-0.3\pm 1.1^{\circ },\text{roll}=0.1\pm 1.4^{\circ }$, and $\text{yaw}=-0.1\pm 0.7^{\circ }$, with 7% of the rotations being $>2^{\circ }$ (Table 1 and Fig. 2). Population systematic error (Σ) in vertical, longitudinal, and lateral directions was 0.19, 0.32, and 0.2 mm. The population random error (σ) in the corresponding directions was 0.37, 0.30, and 0.29 mm. The obtained safety margins by Van Herk's formula were 0.6 cm, 0.8 cm, and 0.6 cm for vertical, longitudinal, and lateral, respectively; these are included in our standard treatment margins. Eighty‐one percent of the translational shifts were <5mm and 93% of the rotations were $<2^{\circ }$.

**Table 1:** Geometrical results from 789 translational and 789 rotational real patient displacements

	*Translation*	*Rotational*	*Lat (mm)*	*Long (mm)*	*Vrt (mm)*	*Pitch (°)*	*Roll (°)*	*Yaw (°)*
Shift>5 mm	19%		13%	24%	19%			
$\text{Shift}>2^{\circ }$		7%				7%	15%	1%
Mean±SD			0.5±3.6	−1.4±4.2	−1.4±4.1	−0.3±1.1	0.1±1.4	−0.1±0.7

The translational shift was observed above all in vertical axis; the difference is considered to be statistically significant (p<0.001) between lateral and the other two axes. Regarding rotational shifts, comparing mean±SD, the pitch ones were the greatest with $-0.3\pm 1.1^{\circ }$ (range 2.2° and $-2.8^{\circ }$), compared with the other two rotations (p<0.05), even if the biggest range rotation was observed in the roll ones (4.0° and $-3.8^{\circ }$) No correlation was observed between the magnitude of translational and rotational shift (all R values<0.2).

### B. Dosimetric analysis

The dosimetric analysis was performed considering 7/12 patients, in which we collected CBCT scans according to the study's schedule, and 172 CBCT out of 175 planned. Three CBCTs were kept out for the analysis because of ring artifact.^(15)^ Two significant linear correlations were observed (3, 4, 5): yaw rotation vs. the V20 right femoral heads (correlation factor r=0.82), yaw rotation vs. the V20 left femoral heads (r=0.72), and pitch rotation vs. V50 rectum (r=−0.65) (p<0.01).

Rototranslational correction seems to impact more on PTV2 (C=0.2±1.3) than on PTV1 coverage (C=0±0.2) (6, 7) (p=0.05). If there is no correction for rotations, in the worst case the target coverage of PTV1 in terms of V95% will still be 97.3% and the target coverage of PTV2 in terms of V95% will be 94.6%.

Analyzing data regarding the replanning phase, in all patients we observed a median reduction of new PTV of 47.83 cc (range 85.6–12.4) and 2.47 cc (range 10 and 1.89) of PTV1 and PTV2, respectively. In only one case the new PTV2 was larger than PTV2 of 5.91 cc.

Comparing Cpl with Crepl (Table 2), despite margin reduction, there is not a significant dosimetric impact of a 6 DOF position correction on PTV1 coverage. On the contrary, the dosimetric impact on PTV2 coverage is more significant during the replanning phase (Cpl=0.1% vs. C repl=0.5%). This observation underlines the need of 6 DOF correction in a margin reduction approach.

**Figure 3:**
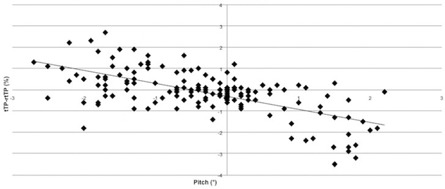
Linear correlation between pitch and V50 rectum.

**Figure 4:**
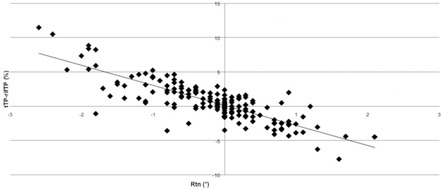
Linear correlation between yaw and V20 right femoral heads.

**Figure 5:**
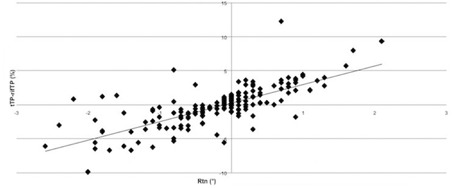
Linear correlation between yaw and V20 left femoral heads.

**Figure 6:**
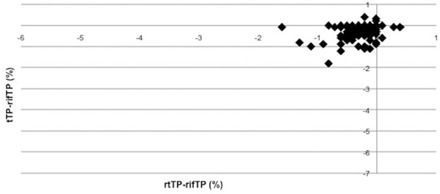
Trend of difference between tTP‐refTP and rtTP‐refTP on PTV1 V95% coverage.

**Figure 7:**
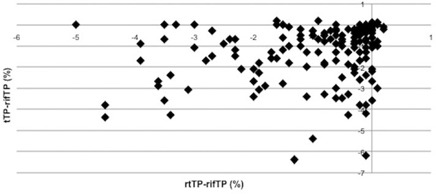
Trend of difference between tTP‐refTP and rtTP‐refTP on PTV2 V95% coverage.

**Table 2:** Dosimetric impact on PTV1 and PTV2 V95% coverage, comparing planning and replanning phases

	*Planning*			*rePlanning*		
	Apl(tTP−refTP)%	Bpl(rtTP−refTP)%	Cpl(Apl−Bpl)	A repl(tTP−replTP)%	B repl(rtTP−replTP)%	C repl(Arepl−Brepl)
PTV1 V95%						
mean	−0.3	−0.2	0.1	−0.3	−0.2	0.1
SD	0.4	0.3	0.1	0.3	0.2	0.1
max	0.4	0.4	0	0.0	0.1	0.1
min	−1.8	−1.6	0.2	−1.1	−1.1	0
PTV2 V95%						
mean	−0.7	−0.8	0.1	−1.6	−1.1	0.5
SD	1.0	1.0	0	1.4	1.4	0
max	0.2	0.2	0	0.2	0.1	0.1
min	−4.3	−3.5	0.8	−6.4	−8.9	2.5

## IV. DISCUSSION

The knowledge of treatment delivery uncertainties is essential in RT planning, especially in the case of IMRT or VMAT technique. The modern linacs offer the possibility of three‐dimensional (3D) volume imaging of the patient in the treatment position using a kilovoltage CBCT scanner. Regular CBCT studies performed before treatment permit to verify patient setup and target position.

Most of the studies which investigated this point reported only translational errors,^27^, ^28^, ^29^, ^30^ generally ranging between 0.5 and 1.8 mm.^(14)^ Limited data exist regarding the magnitude and relevance of rotational errors or correlation between translational and rotational shift. But when rotational deviation is greater than 2° or target's longitudinal diameter is longer than 3 cm from the isocenter, the translational deviation is more than could be significant if a tight margin is used.^10^, ^14^ Some authors report variations in prostate rotations $<2^{\circ }$ ^15^, ^31^, ^32^ with pitch greater than yaw or roll. Our results are comparable with translational shift ranged between 0.5 to 1.4 mm, 7% of rotational shift $>2^{\circ }$, with the higher systematic error in pitch. The reason might be that the rotations around vertical and longitudinal axes can be controlled quite well by careful alignment of the left and right skin markers and using the symmetry of the patient. This reference is not available for rotation adjustments around the lateral axis where only the anterior marker can be used. Furthermore, the position of the leg and the use of a knee support may also cause the lateral rotational setup errors. This is the first geometrical and dosimetric analysis on prostate VMAT treatment plan using Protura Robotic Patient Positioning System, reconstructing and not simulating prostate delivered dose through the real daily deviations. Our geometric results (translational shift <5mm in 81% of patients and rotational shift $<2^{\circ }$ in 93% of cases) are comparable with those reported by other authors^14^, ^15^, ^33^ with a larger translational SD or random errors, possibly related to patient's compliance or organ motion. Regarding rotational displacement, the mean systematic errors, ranging from 0.1° to 0.3°, and the random error, ranging from 0.7° and 1.1°, compare respectively with those reported by Guckenberger et al.^(14)^ and Van Herten et al.^(34)^ Also in our experience, no correlation was seen between the magnitude of the translational and rotational shift. The relevance of rotational and translational errors is not necessarily equivalent or proportional. We observed large rotational errors even in the case of small translational errors; this supports the benefits of 3D imaging and of the 6 DOF robotic couch device.

Several authors studied the target coverage of the prostate after applying translational and rotational position correction. A no significant impact on the dosimetric target coverage in prostate cancer was reported.^11^, ^35^, ^36^, ^37^, ^38^ Van Haaren et al.^(15)^ have shown that when there is no correction for rotations, the target coverage of the prostate is still 98.7% and 95.7% for the seminal vesicles, even though the planning was done without PTV margin. Therefore, they concluded that prostate pitch rotations have minimal impact on prostate coverage given that translations were managed. This conclusion was based on evaluating dosimetric impact of prostate rotations using simulated IMRT treatment plans for five patients with different rotation errors and using no margin around CTV as the best situation to study the effect of rotations and corrections. Orton and Tomé^(39)^ neglected rotations of the prostate and found that the dose distribution after applying position correction was nearly identical to the treatment plan.

On the contrary Amro et al.,^(32)^ using an average of real‐time tracking 6D displacement and rigidly translating and rotating the prostate (as we did in the present study), retrospectively evaluated PTV expansion of 0, 2, 3, 5 mm and showed that rotation can still have a substantial impact on prostate delivered dose, but probably on specific patients.

Our experience shows that the dosimetric impact is related to several factors. In the present study, contrary to previously published studies, we used real daily interfraction displacements (789 translational and 789 rotational errors) and the estimated delivered dose was analyzed on 344 VMAT‐SIB TPs. The impact of 6D correction was not significant on dosimetric prostate coverage, while it did influence the seminal vesicles coverage. The different impact might be due to the almost spherical shape of the prostate, and to the seminal vesicles elongated shape which are more sensitive to rotation errors.^(11)^ A more relevant dosimetric impact is present in the replanning phase, characterized by a margin reduction. Following these observations, it is better to regularly correct and make the systematic error as small as possible in clinical practice, especially in a margin reduction approach.

Regarding critical structures, we observed that femoral heads are more sensitive to yaw rotation. In fact, on the robotic couch the whole body rotates and a rotation is introduced especially for structure distant from the rotation point. Therefore, the shape and the distance from isocenter have a role in the dosimetric significance, as reported also for stereotactic technique. ^40^, ^41^ Using real daily shift approximates the dose calculations to the clinical reality, although not all factors are taken into account because of rigid registration. Also Van Herten et al.^(34)^ assumed prostate, bladder, and rectum as rigid structures, considering that deformations are not fully visible when using gold markers for position verification. Internal deformation of the prostate can generally be neglected, and both bladder and rectum can be mobile, especially concerning shrinkage and expansion. But we preferred reliable imaging registration algorithm in this exploratory phase of robotic couch implementation.^(42)^

In the present study, interfraction displacements were considered. The next ongoing step is the reduction of the frequency of acquisition to perform a secondary CBCT after the 6D correction to investigate the possible patient motion induced by automated couch movement during on line repositioning, as already hypothesized,^3^, ^29^, ^43^ especially when large rotations (pitch and roll) are to be corrected.^(44)^ In fact, it has been suggested that couch corrections prior to treatment may result in further positioning.

Errors,^7^, ^10^, ^44^, ^45^, ^46^, ^47^ especially when there are large rotations (tilt and roll), are to be corrected. ^(44)^ A strategy has been proposed by some authors^(43)^ for the use of the robotics system, such as using the first five treatment fractions as a predictor for the patient's behavior during the remaining treatment fractions, but, from their experience in prostate cancer, the 6D setup correction does not induce secondary motion in most of patients, maybe due to the proximity of the prostate to the rotation center. The real‐time intrafractional variation will be considered in further analysis.

## V. CONCLUSIONS

In our experience the Protura System was user friendly, provided for a fast learning curve, and integrated naturally into our daily clinical practice with no changes to our IGRT workflow. This allows us to investigate the relevance of the geometrical uncertainties for other treatment indications (e.g., brain, H&N, lung, spinal cord) and also for other technique such as 3D CRT, IMRT, and SBRT. No correlation was observed between translational and rotational errors in prostate treatment.

Dosimetric relevance of 6D patient repositioning is shown in the case of nonspherical elongated targets, such as vesicles, or when critical structures are distant from the isocenter, as femoral heads, or adjacent to sharp dose gradient, as the rectum. A study considering the impact of intrafractional error, which also defines the margins for VMAT prostate treatment ensuring the target coverage, is under way. Furthermore, the deformable registration will be validated to calculate the realistic dose received from target and critical structures.
